# Real-World Analysis of the Aging Effects on Visual Field Reliability Indices in Humans

**DOI:** 10.3390/jcm10245775

**Published:** 2021-12-09

**Authors:** Tomoki Shirakami, Tetsuro Omura, Hiroki Fukuda, Ryo Asaoka, Masaki Tanito

**Affiliations:** 1 Department of Ophthalmology, Faculty of Medicine, Shimane University, Izumo 693-8501, Japan; t-shira@med.shimane-u.ac.jp (T.S.); omuomu@med.shimane-u.ac.jp (T.O.); hiroki17@med.shimane-u.ac.jp (H.F.); 2 Department of Ophthalmology, Graduate School of Medicine, University of Tokyo, 7-3-1 Hongo, Bunkyo-ku, Tokyo 113-8655, Japan; rasaoka-tky@umin.ac.jp; 3 Department of Ophthalmology, Seirei Hamamatsu General Hospital, Shizuoka 430-8558, Japan; 4 Seirei Christopher University, Shizuoka 422-8545, Japan; 5 Nanovision Research Division, Research Institute of Electronics, Shizuoka University, Shizuoka 422-8529, Japan; 6 The Graduate School for the Creation of New Photonics Industries, Shizuoka 431-1202, Japan

**Keywords:** visual field, glaucoma, aging, real-world data, fixation loss, FL, false negative, FN, false positive, FP

## Abstract

Relationships between age and visual field (VF) reliability indices were investigated using a large real-world dataset (42,421 VF data points from 11,525 eyes of 5930 subjects). All VFs tested and stored at Shimane University Hospital between 1988 and 2019 were exported. Correlations between age, mean deviation (MD), pattern standard deviation (PSD), and reliability indices including fixation losses (FLs), false negatives (FNs), and false positives (FPs) were analyzed. The mean ± standard deviation age was 65.0 ± 15.1 years; MD—−6.9 ± 8.1 decibels (dB); PSD—6.3 ± 4.6 dB; FL—8.6 ± 11.7%; FN—5.3 ± 8.3%; and FP—2.6 ± 5.0%. Univariate analyses showed strong associations between age and FNs (correlation coefficient, ρ = 0.20, *p* < 0.0001) and MD (ρ = −0.21, *p* < 0.0001). All FLs, FNs, and FPs were lowest during the third decade (20–29 years) of life. FLs were elevated consistently after that decade, and FNs were elevated sharply after the seventh decade. FPs were relatively stable after the fourth decade (30–39 years). Mixed-effect regression analyses in subjects 40 years and older showed that older age was associated with worse FLs (*p* < 0.0001) and FNs (*p* < 0.0001) but not FPs (*p* = 0.4126). Aging affects FLs and FNs with different modes but had minimal effects on FPs. Decreased VF sensitivity, deteriorated macular function, and technical difficulties with testing may be mechanisms of age-related changes in FLs and FNs.

## 1. Introduction

The use of semiautomated perimeters in ophthalmology is essential, especially in the management of patients with glaucoma. Early detection of visual field (VF) progression and determining the estimated progression rate are important for managing glaucoma [1]. Retinal function is assessed by determining light sensitivity thresholds using static perimetry methods. Test results are presented quantitatively, which is particularly suitable for interpretating the VF data using statistical and other less subjective methods of analysis. Many studies have shown that the presence and severity of glaucoma are related to higher variability [2,3,4,5,6]. A few studies have indicated other patient-related factors associated with VF fluctuation, including visual acuity [7], ethnicity [8], and cognitive decline [9]. Jaffe et al. reported that age-related decline in threshold sensitivity and the standard error of the decline increased with fixation eccentricity [10]. It is important to determine how normal VF is affected by factors such as retesting, fatigue, and aging. Drance et al. reported that the area of kinetic perimetry decreased with age [11]. Similarly, the normal standards published for OCTOPUS perimetry include adjustments derived from a measured linear decline in light sensitivity as an aging function [10]. Not many studies have provided a comprehensive evaluation of reliability of perimetry tests associated with aging. In this study, we investigated the relationship between age and reliability indices such as fixation losses (FLs), false negatives (FNs), and false positive (FPs) from a large dataset of perimetry tests.

## 2. Subjects and Methods

### 2.1. Subjects

The study adhered to the tenets of the Declaration of Helsinki; the institutional review board (IRB) of Shimane University Hospital reviewed and approved the research (study no. 20080911-1). IRB approval did not require that each patient provide written informed consent for publication; instead, the study protocol was posted at the study institutions to notify participants about the study. In order to perform real-world analysis, all VF data obtained during 42,421 VF tests using the Central 30-2 program Humphrey Visual Field Analyzer (Carl Zeiss Meditec, Dublin, CA, USA), tested with SITA-standard (82%) or full-threshold program (18%), and stored at the Department of Ophthalmology, Shimane University Hospital, between 1988 and 2019 were exported. These VF data, which included 199 cases in which patient identification was unknown, comprised 11,525 eyes of 5930 Japanese subjects (mean age ± standard deviation [SD], 65.1 ± 15.1 years). For analyses, subjects’ identification, age at VF testing, mean deviation (MD), pattern standard deviation (PSD), and rates of FL, FNs, and FPs were collected.

### 2.2. Statistical Analysis

The data were expressed as the means ± SD for continuous variables. Possible correlations among age and VF indices (MD, PSD, FL, FN, and FP) were assessed using the Spearman’s rank correlation test. VF reliability parameters (FL, FN, and FP) were compared between age groups stratified by 10-year increments (i.e., 0–9 years, 10–19 years, and 90–99 years) by one-way analysis of variance followed by the post-hoc Tukey honesty significant difference (HSD) test for the adjustment of multi-pair comparisons. In order to further assess the effect of aging on VF reliability, mixed-effect regression analyses were performed in subjects 40 years or older with age, MD, and PSD set as the fixed effect and subject identification and tested eye (right or left) set as the random effect. All statistical analyses were calculated using JMP Pro statistical software version 14.2 (SAS Institute, Inc., Cary, NC, USA).

## 3. Results

**Figure 1:**
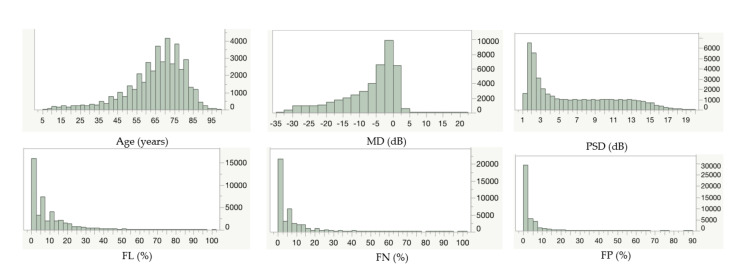
Distribution of the parameters. MD, mean deviation; PSD, pattern standard deviation; dB, decibel; FL, fixation loss; FN, false negative; FP, false positive.

Subject demographic data and their distributions are shown in Table 1 and Figure 1, respectively. The maximum value of MD was + 20.47; this data was obtained from a 7-year-old child, with 23/28 poor fixation and 50% of FP.

Table 2 shows the possible association among age and VF parameters. All combinations of each parameter except for the pairs of PSD-FL and PSD-FP were correlated significantly (*p* < 0.0001); strong correlation was observed between age and FN (correlation coefficient, ρ = 0.20) or MD (ρ = −0.21).

**Figure 2:**
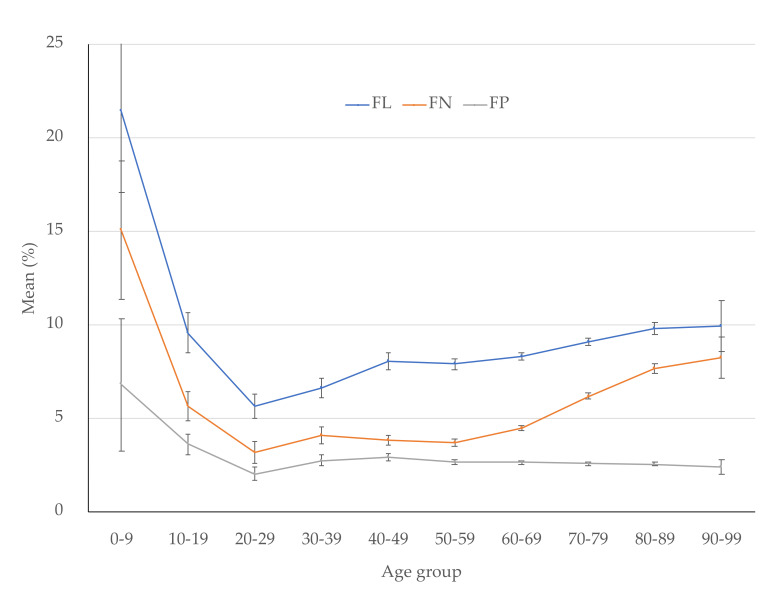
Mean values of FLs, FNs, and FPs in each age-stratified group. FL, fixation loss; FN, false negative; FP, false positive. Error bars indicate 95% confidence interval.

Table 3, Table 4 and Table 5 show FLs, FNs, and FPs, respectively, in each age-stratified group. FLs, FNs, and FPs were significantly lower in patients in the third decade of life compared with other age groups other than the fourth decade of life regarding FLs and FNs. FLs were elevated consistently after the third decade of life, and FNs were elevated sharply after the seventh decade of life, while FPs were relatively stable after the fourth decade of life (Figure 2).

The effect of age on reliability indices was assessed further in subjects 40 years and older by mixed-effect regression analyses to adjust for possible confounding effects derived from the difference in severity of VF defects and from the inclusion of repeated measurements or both eyes of a subject (Table 6). Older age was significantly associated with worse FLs (*p* < 0.0001) and FNs (*p* < 0.0001) but not with FPs (*p* = 0.4126).

## 4. Discussion

We analyzed the relationship between age and reliability indices in a large dataset of perimetry tests. We found that all FLs, FNs, and FPs were lowest in subjects in the third decade of life, FLs were elevated consistently after the third decade of life, FNs were elevated sharply after the seventh decade of life, and FPs were relatively stable after the fourth decade of life. In addition, FLs and FNs were associated with aging, while FPs were not.

VF testing is a probabilistic rather than deterministic examination [12]. Due to the fact that VF testing is intrinsically variable, a certain amount of random variability exists even in healthy, trained, and reliable participants [13,14]. The variability results in worse reliability indices of VF testing, which is an obstacle to the accurate quantification of VF progression. The minimal number of VF assessments required to detect fast progressors (MD rate, –1 dB/year) based on the MD rate of change may range from 6 to 13 depending on the degree of variability [15]. Patients with large VF fluctuations may benefit from frequent testing and the use of more sophisticated and sensitive methods to detect progression to reduce the time required to identify progression [1,16,17,18]. Therefore, reliability indices are important for assessing VF testing and managing glaucomatous patients, which has been reported previously [13,19,20,21,22,23,24]. We investigated the relationship between reliability indices and aging, which is one of the most influential factors.

Considering the data in Table 3 and Table 6, FLs and FNs are associated with aging, but FPs are not. In addition, transitions of FLs and FNs differ; FLs are elevated consistently after the third decade of life, and FNs are sharply elevated after the seventh decade of life, which may result from different visual function changes, e.g., FL is related to macular function [25,26]. Another factor may be technical difficulties during testing because of age-related changes such as decreased cognitive function and/or physical disability. This possibility is more reasonable because patients with poor fixation can maintain their fixation more easily with Goldmann perimetry rather than the Humphrey Field Analyzer [27]. According to our study, FNs were related to MD and aging. The relationship between FNs and MD was suggested previously [2,6]. Russell et al. reported that MD variability increases with increasing damage [3]. It also has been reported that FN frequencies were higher in eyes with field loss such as in glaucomatous eyes, which may be explained by increased variability in threshold values typically found in such eyes [28]. However, few reports assessed the relationship between FNs and aging especially in normal eyes. Adams et al. reported that after age 70 years, there was a slightly greater sensitivity loss with age in normal eyes [29]. Therefore, glaucoma and aging both cause VF sensitivity loss, which may be related to age-dependent changes in FNs more remarkable than FPs. Our study also found a strong association between age and MD. This may be explained by the progression/acceleration of diseases by aging (e.g., glaucoma) [30].

The limitation of this study was the absence of the consideration of some physical factors such as fatigue and loss of concentration, which may affect the results of reliability indices in that the eye tested second has a greater amount of variability [31,32,33]. However, these studies were based on older, prolonged examination protocols that are no longer used in clinical practice. The effect of eye testing order on VF variability with SITA algorithms has been investigated in a few studies with controversial results [33,34,35]. The absence of clinical backgrounds such as visual acuity and ocular pathology is the limitation of the current study. However, we believe that the study design is reasonable for assessing the overall impact of age on the reliability indices of VF testing.

## 5. Conclusions

This study described the relationships between ages and each reliability indices; FLs are consistently elevated after the third decade of life, and FNs elevated sharply after the seventh decade, while FPs were relatively stable after the third decade of life. These changes are thought to have resulted from the decline in VF sensitivity, macular function deterioration, and technical difficulties that were tested, which were caused by aging.

## Figures and Tables

**Table 1:** Demographic subject data.

Parameters	Mean ± SD	Range
Age (years)	65.0 ± 15.1	5–99
MD (dB)	−6.9 ± 8.1	−34.51–20.47
PSD (dB)	6.3 ± 4.6	1–19.78
FL (%)	8.6 ± 11.7	0–100
FN (%)	5.3 ± 8.3	0–100
FP (%)	2.6 ± 5.0	0–89

SD, standard deviation; MD, mean deviation; PSD, pattern standard deviation; dB, decibel; FL, fixation loss; FN, false negative; FP, false positive.

**Table 2:** Possible associations among parameters.

	ρ *	Age (years)	MD (dB)	PSD (dB)	FL (%)	FN (%)	FP (%)
*p* *							
Age (years)			−0.21	0.17	0.08	0.20	0.05
MD (dB)		<0.0001 *		−0.82	0.06	−0.32	0.12
PSD (dB)		<0.0001 *	<0.0001 *		−0.00	0.31	0.00
FL (%)		<0.0001 *	<0.0001 *	0.7688		0.15	0.30
FN (%)		<0.0001 *	<0.0001 *	<0.0001 *	<0.0001 *		0.10
FP (%)		<0.0001 *	<0.0001 *	0.3553	<0.0001 *	<0.0001 *	

The correlation coefficient (ρ) and *p* values are calculated using Spearman’s rank correlation test. * *p* < 0.0001. MD, mean deviation; PSD, pattern standard deviation; dB, decibel; FL, fixation loss; FN, false negative; FP, false positive.

**Table 3:** Comparison of FLs among age-stratified groups.

Age Group (Years)	No.	Mean (%)± SD	Lower 95% CI	Upper 95% CI	*p*-Value								
					vs. 10–19	vs. 20–29	vs. 30–39	vs. 40–49	vs. 50–59	vs. 60–69	vs. 70–79	vs. 80–89	vs. 90–99
0–9	63	21.43 ± 17.32	17.07	25.80	<0.0001 *	<0.0001 *	<0.0001 *	<0.0001 *	<0.0001 *	<0.0001 *	<0.0001 *	<0.0001 *	<0.0001 *
10–19	624	9.55 ± 13.49	8.49	10.61		<0.0001 *	<0.0001 *	0.0977	0.0232 *	0.2199	0.9912	0.9999	1.0000
20–29	858	5.64 ± 10.13	4.96	6.32			0.6482	<0.0001 *	<0.0001 *	<0.0001 *	<0.0001 *	<0.0001 *	0.0008 *
30–39	1340	6.63 ± 9.47	6.12	7.13				0.0071*	0.0128 *	<0.0001 *	<0.0001 *	<0.0001 *	0.0004 *
40–49	3063	8.05 ± 12.39	7.61	8.49					0.9997	0.9868	0.0007 *	<0.0001 *	0.1903
50–59	6112	7.88 ± 11.8	7.59	8.18						0.3904	<0.0001 *	<0.0001 *	0.0889
60–69	11376	8.31 ± 11.12	8.10	8.51							<0.0001 *	<0.0001 *	0.3396
70–79	12899	9.06 ± 11.62	8.86	9.26								0.0017 *	0.9590
80–89	5749	9.82 ± 12.46	9.50	10.14									1.0000
90–99	296	9.94 ± 11.78	8.59	11.28									

To adjust multi-pair comparisons, the *p*-values are calculated using Tukey–Kramer’s honestly significant difference test between each pair of groups. * *p* < 0.05. SD, standard deviation; CI, confidence interval; FL, fixation loss.

**Table 4:** Comparison of FNs among age-stratified groups.

Age Group (Years)	No.	Mean (%) ± SD	Lower 95% CI	Upper 95% CI	*p*-Value								
					vs. 10–19	vs. 20–29	vs. 30–39	vs. 40–49	vs. 50–59	vs. 60–69	vs. 70–79	vs. 80–89	vs. 90–99
0–9	63	15.07 ± 14.69	11.37	18.77	<0.0001 *	<0.0001 *	<0.0001 *	<0.0001 *	<0.0001 *	<0.0001 *	<0.0001 *	<0.0001 *	<0.0001 *
10–19	624	5.66 ± 9.78	4.89	6.42		<0.0001 *	0.0032 *	<0.0001 *	<0.0001 *	0.0139 *	0.8608	<0.0001 *	0.0006 *
20–29	837	3.2 ± 8.67	2.61	3.79			0.2796	0.6476	0.8105	0.0006 *	<0.0001 *	<0.0001 *	<0.0001 *
30–39	1305	4.09 ± 8.42	3.63	4.55				0.9898	0.8616	0.8660	<0.0001 *	<0.0001 *	<0.0001 *
40–49	3040	3.81 ± 6.83	3.57	4.05					0.9998	0.0036 *	<0.0001 *	<0.0001 *	<0.0001 *
50–59	5997	3.7 ± 6.67	3.53	3.87						<0.0001 *	<0.0001 *	<0.0001 *	<0.0001 *
60–69	11164	4.46 ± 7.41	4.33	4.60							<0.0001 *	<0.0001 *	<0.0001 *
70–79	12310	6.18 ± 8.87	6.02	6.34								<0.0001 *	0.0018 *
80–89	5239	7.67 ± 9.43	7.42	7.93									0.9839
90–99	267	8.24 ± 9.4	7.11	9.37									

To adjust multi-pair comparisons, the *p*-values are calculated using Tukey–Kramer’s honestly significant difference test between each pair of groups. * *p* < 0.05. SD, standard deviation; CI, confidence interval; FN, false negative.

**Table 5:** Comparison of FPs among age-stratified groups.

Age Group (Years)	No.	Mean (%) ± SD	Lower 95% CI	Upper 95% CI	*p*-Value								
					vs. 10–19	vs. 20–29	vs. 30–39	vs. 40–49	vs. 50–59	vs. 60–69	vs. 70–79	vs. 80–89	vs. 90–99
0–9	63	6.78 ± 14.11	3.23	10.34	<0.0001 *	<0.0001 *	<0.0001 *	<0.0001 *	<0.0001 *	<0.0001 *	<0.0001 *	<0.0001 *	<0.0001 *
10–19	635	3.61 ± 7.19	3.05	4.17		<0.0001 *	0.0106 *	0.0389 *	0.0004 *	<0.0001 *	<0.0001 *	<0.0001 *	0.0223 *
20–29	860	2.03 ± 5.14	1.69	2.37			0.0342 *	0.0002 *	0.0096 *	0.0180 *	0.0641	0.1045	0.9785
30–39	1340	2.74 ± 5.46	2.45	3.03				0.9911	1.0000	0.9995	0.9687	0.9670	0.9911
40–49	3073	2.91 ± 6.03	2.69	3.12					0.6140	0.1945	0.0226 *	0.0491 *	0.8354
50–59	6115	2.69 ± 5.11	2.56	2.82						0.9998	0.851	0.9027	0.9956
60–69	11383	2.64 ± 5.22	2.54	2.73							0.9807	0.9892	0.9990
70–79	12902	2.57 ± 4.46	2.49	2.64								1.0000	1.0000
80–89	5751	2.55 ± 3.98	2.45	2.66									1.0000
90–99	296	2.42 ± 3.49	2.02	2.81									

To adjust multi-pair comparisons, the *p*-values are calculated using Tukey–Kramer’s honestly significant difference test between each pair of groups. * *p* < 0.05. SD, standard deviation; CI, confidence interval; FP, false positive.

**Table 6:** Possible association between each reliability index and various parameters in subject 40 years and older using a mixed-effect model.

Reliability Index	FL (%)			FN (%)			FP (%)		
Parameters	R	95% CI	*p*-Value	R	95% CI	*p*-Value	R	95% CI	*p*-Value
Age (/years)	0.05	0.03–0.06	<0.0001 **	0.04	0.03–0.06	<0.0001 **	0.00	−0.00–0.01	0.4126
MD (/dB)	0.16	0.14–0.18	<0.0001 **	−0.29	−0.32–−0.29	<0.0001 **	0.14	0.13–0.15	<0.0001 **
PSD (/dB)	0.12	0.08–0.16	<0.0001 **	0.13	−0.10–0.16	<0.0001 **	0.14	0.12–0.15	<0.0001 **

Subject identification is adopted as a random effect. *p*-values are calculated by multiple regression analysis. ** *p* < 0.01. FL, fixation loss; FN, false negative; FP, false positive; R, regression coefficient; CI, confidence interval; MD, mean deviation; dB, decibel; PSD, pattern standard deviation.

## Data Availability

Data are fully available upon reasonable request to the corresponding author.
